# Clinical Significance of the Interleukin 24 mRNA Level in Head and Neck Squamous Cell Carcinoma and Its Subgroups: An In Silico Investigation

**DOI:** 10.1155/2020/7042025

**Published:** 2020-09-18

**Authors:** Lan-Lan Qiu, Xiao-Guohui Zhang, Gang Chen, Yi-Wu Dang, Zhi-Guang Huang, Ming-Xuan Li, Yao Liang, Su-Ning Huang, Xiao-Zhun Tang, Xiao-Xia Chen, Hong-Yu Wei, Hua-Yu Wu

**Affiliations:** ^1^Department of Cell Biology and Genetics, School of Pre-clinical Medicine, Guangxi Medical University, Nanning 530021, Guangxi Zhuang Autonomous Region, China; ^2^Department of Pathophysiology, School of Pre-clinical Medicine, Guangxi Medical University, Nanning 530021, Guangxi Zhuang Autonomous Region, China; ^3^Department of Pathology, First Affiliated Hospital, Guangxi Medical University, Nanning 530021, Guangxi Zhuang Autonomous Region, China; ^4^Department of Head and Neck Tumor Surgery, Guangxi Medical University Cancer Hospital, Nanning 530021, Guangxi Zhuang Autonomous Region, China; ^5^Department of Radiotherapy, Guangxi Medical University Cancer Hospital, Nanning 530021, Guangxi Zhuang Autonomous Region, China; ^6^Department of Organic Chemistry and Medicinal Chemistry, Pharmaceutical College, Guangxi Medical University, Nanning 530021, Guangxi Zhuang Autonomous Region, China

## Abstract

IL24 mRNA is known to have an apoptotic effect on cancer cells but not on noncancer cells. However, the expression level of the IL24 mRNA in head and neck squamous cell carcinoma (HNSCC) and its subgroups is rarely studied. In this study, the clinical implication of IL24 mRNA was evaluated in the common subgroups of HNSCC, including oral squamous cell carcinoma (OSCC), nasopharyngeal carcinoma (NPC), and laryngeal squamous cell carcinoma (LSCC) for analysis. Substantial IL24 mRNA expression data were calculated from several databases, such as the Gene Expression Omnibus (GEO), ArrayExpress, Sequence Read Archive (SRA), ONCOMINE, and The Cancer Genome Atlas (TCGA) databases. We ultimately collected a total of 41 microarrays and RNA-seq including 1,564 HNSCC and 603 noncancer tissue samples. IL24 mRNA was highly expressed in OSCC, LSCC, and NPC as shown by the separated standard mean difference (SMD), as well as HNSCC as a whole part (SMD = 1.47, 95% confdence interval (CI) = 1.24−1.70, *P* < 0.0001). In all subgroups, the IL24 mRNA upregulation had the ability to distinguish cancer from noncancer tissue with area under the curves (AUCs) of the summary receiver operating characteristic (sROC) higher than 0.85. In conclusion, IL24 mRNA may be used as a potential marker for cancer screening, and its clinical diagnostic value needs to be further studied. It also provides a new idea for the treatment of the IL24 gene in HNSCC and its subgroups in the future.

## 1. Introduction

Head and neck squamous cell carcinoma (HNSCC) is the sixth most common cancer in the world and has a five-year survival rate of less than 50% [1, 2]. HNSCC has high metastasis and recurrence rates and includes the following subgroups: oral squamous cell carcinoma (OSCC), nasopharyngeal carcinoma (NPC), and laryngeal squamous cell carcinoma (LSCC) [2, 3]. Similar to other cancers, HNSCC is caused by abnormal genetic changes, such as point mutations, amplifications, gene rearrangements, and deletions, which can promote the development of tumors [4]. HNSCC is also associated with a variety of environmental factors as known risk factors, including smoking, alcohol abuse, and human papillomavirus (HPV) infection [5]. The survival rate of patients with the disease has increased due to progress in surgical therapy, as well as radiotherapy and chemotherapy. In addition, immunotherapy has become a promising treatment for HNSCC [6, 7]. However, due to the lack of obvious early clinical symptoms, many HNSCC patients are diagnosed with advanced cancer. Moreover, the prognosis of HNSCC patients remains stagnant, with a considerable number of deaths due to recurrence and metastasis after chemotherapy and targeted therapy [8]. Furthermore, there are no suitable tumor markers for early diagnosis and prognosis evaluation of HNSCC [4].

The Interleukin 24 mRNA (IL24 mRNA), a single copy gene, is located at chromosome 1q32-41. IL24 is expressed as a cytokine in a variety of immune tissues and cells at normal physiological levels. At the supraphysiological level, IL24 mRNA significantly inhibits tumor growth, invasion, metastasis, and angiogenesis [9]. It is a polyergic immunoregulatory cytokine that belongs to the IL-10 gene family, with a wide range of antitumor properties, including specific induction of tumor cell apoptosis, inhibition of tumor angiogenesis, and regulation of antitumor immune responses [10, 11]. Other studies have shown that IL24 mRNA can enhance the immunogenicity of tumor cells through upregulation of costimulatory molecules, such as CD80 and CD86 [12]. Some studies show that IL24 mRNA can inhibit several types of cancer cells, such as prostate cancer, kidney cancer, osteosarcoma, melanoma, colon cancer, cervical cancer, malignant glioma, breast cancer, and lung cancer, without obvious adverse effects on normal cells [13]. Recent evidence indicates that the IL24 mRNA is an underlying candidate for gene treatment of cancer through boosting apoptosis in many types of cancer cells [14–17]. IL24 has been reported to play vital roles in the inhibition of tumor growth and induction of tumor cell apoptosis in HNSCC cells [18, 19]. Adenovirus-mediated IL24 mRNA therapy could also enhance the in vivo and in vitro antitumor effects of radiotherapy for NPC [20, 21].

However, the literature retrieved above only discussed the treatment of HNSCC and its subgroups with IL24 mRNA in cell lines or animals, and the current studies have not further analyzed the safety assessment and clinical treatment trials as a feasible means. The expression of the IL24 mRNA in HNSCC and its subgroups and the ability of the IL24 mRNA to distinguish cancer tissue from noncancer tissue are rarely studied. Therefore, we collected 41 data sets of microarrays and RNA-Seq from several databases and speculated that there may be a close relationship between IL24 mRNA expression and HNSCC. In this study, we analyzed IL24 mRNA expression in HNSCC and its subgroups, OSCC, LSCC, and NPC, as well as the corresponding clinical parameters. The results showed that a high expression level of the IL24 mRNA correlated with HNSCC and its subgroups to a certain extent, which potentially has diagnostic ability and therapeutic significance and could be further studied as a potential factor in the future.

## 2. Materials and Methods

### 2.1. Mining the RNA-Seq Data of the IL24 mRNA Based on the Cancer Genome Atlas (TCGA) Database

**Figure 1:**
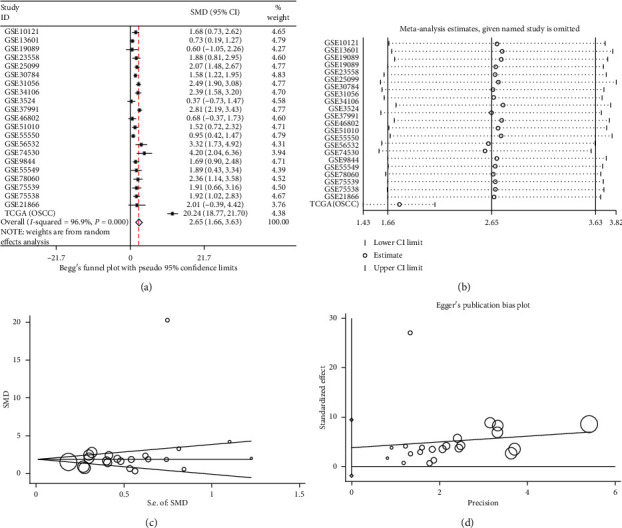
(a) The forest plot of 22 data sets evaluating the standard mean difference (SMD) of Interleukin 24 (IL24) mRNA expression between oral squamous cell cancer (OSCC) tissues and noncancer tissues based on Gene Expression Omnibus (GEO) and The Cancer Genome Atlas (TCGA) databases. (b) Sensitivity analysis of the expression level of IL24 mRNA in the OSCC and noncancer groups. (c) Begg's funnel plot for the publication bias test. (d) Egger's funnel plot for the publication bias test.

After mining the TCGA database, we downloaded the RNA-Seq data of mRNA expression and clinicopathological parameters in HNSCC and extracted data on the IL24 mRNA, which involved 502 cases with HNSCC samples (including 40 cases with tonsil samples, 341 cases with oral cavity samples, 10 cases with hypopharynx samples, and 111 cases with larynx samples) and 44 cases with noncancer samples as a control group. The extracted data were used to analyze the expression and clinical parameters, such as gender, age, and pathological stage, of patients with the IL24 mRNA in HNSCC and its subgroups. The prognostic value of IL24 mRNA was estimated by using the Kaplan–Meier survival analysis.  Figure 1 (in the supplementary materials) shows the data screening and flow chart of the study.

### 2.2. Mining the Microarrays of the IL24 mRNA Based on the Gene Expression Omnibus (GEO) and ArrayExpress Databases

After mining the data from GEO, ArrayExpress, Sequence Read Archive (SRA), and Oncomine databases, we downloaded the microarray data of IL24 mRNA expression in HNSCC and its subgroups and screened the data that conformed to the conditions of this study. The search strategy was (head and neck OR oral OR laryngeal OR nasopharyngeal OR pharyngeal OR HNSCC OR LSCC OR NPC OR OSCC) AND (squamous cell carcinoma OR carcinoma OR tumor OR cancer OR neoplas*∗* OR malignan*∗*). We filtered the retrieved results according to the following inclusion criteria: (1) the species was *Homo sapiens*; (2) samples were from head and neck tissue (including laryngeal, oral, tonsil, tongue, pharyngeal, and nasopharyngeal tissue), blood, and cell lines; (3) the tumor tissues were diagnosed as HNSCC; (4) the expression profiling data of the IL24 mRNA was provided; and (5) the data type was total mRNA.

### 2.3. Statistical Analysis

With SPSS 22.0 software (Chicago, IL, USA), an independent samples *t*-test was used to investigate the difference in IL24 mRNA expression between HNSCC tissues and noncancer tissues based on microarrays and RNA-Seq. Furthermore, the clinical parameters were also calculated to analyze the factors associated with HNSCC. To visualize these calculations, GraphPad Prism Version 7.0 (San Diego, CA, USA) was used to create a box diagram and receiver operating characteristic (ROC) curve for each data set. Moreover, the standard mean difference (SMD) and 95% confidence interval (CI) were utilized to show the different expression levels of the IL24 mRNA between HNSCC tissues and noncancer tissues with Stata 12.0 software (StataCorp, College Station, TX, USA). The results of heterogeneity are reported as a *P* value or Chi-square test results. A *P* value less than 0.05 or *I*^2^ value greater than 50% indicates heterogeneity. Therefore, the random effects model was applied. Otherwise, we used the fixed effects model. In addition, we constructed forest plots to visualize the results. Stata 12.0 was also utilized to construct the summary ROC (sROC) curves to visualize the capability of the IL24 mRNA to distinguish tumor tissues and noncancer tissues, and the area under the curve (AUC) was used to evaluate the accuracy of this capability.

## 3. Results

### 3.1. The Clinical Significance of the IL24 mRNA in OSCC by RNA-Seq

In this study, we downloaded the expression profile of the IL24 mRNA in HNSCC from TCGA database and extracted the expression data and corresponding clinical information for 341 cases with OSCC samples. Compared with the 44 cases with noncancer samples, the OSCC samples showed significantly higher IL24 mRNA expression (*P* < 0.0001, Figure 2(m) in the supplementary materials). In addition, we analyzed the relevant clinical parameter data, but not found the factors which had a statistically significant correlation between the high expression level of the IL24 mRNA and OSCC (Table 1). The Kaplan–Meier curve suggested that IL24 mRNA expression had no significance with the prognosis of the patients (Figure 2(p) in the supplementary materials).

### 3.2. The Analysis of IL24 mRNA Expression in OSCC by Microarrays and RNA-Seq Data

**Figure 2:**
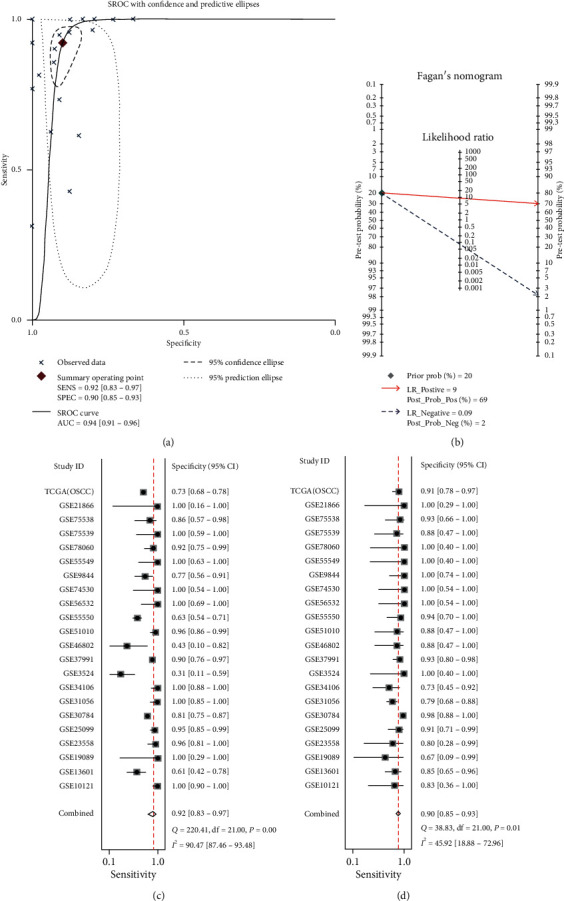
(a) The summary receiver operating characteristic curves analysis of IL24 mRNA for discriminating OSCC from noncancer tissues based on GEO and TCGA databases. (b) Prior probability and postprobability positive and negative of the included studies. The prior probability, postprobability positive, and postprobability negative reached 20%, 69%, and 2%, respectively (LR: likelihood ratio). (c, d) Sensitivity and specificity values of the included studies. The sensitivity and specificity values of the included studies were 0.92 (95%CI = 0.83−0.97) and 0.90 (95%CI = 0.85−0.93), respectively.

**Figure 3:**
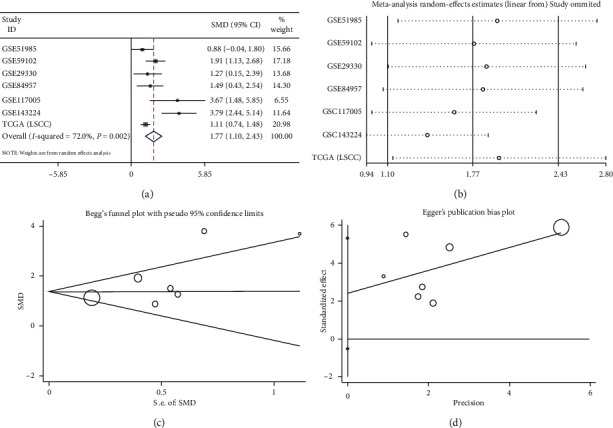
(a) The forest plot of 7 data sets. (b) Sensitivity analysis of analysis of the expression level of IL24 mRNA in the LSCC and noncancer groups. (c) Begg's funnel plot for the publication bias test. (d) Egger's funnel plot for the publication bias test.

**Figure 4:**
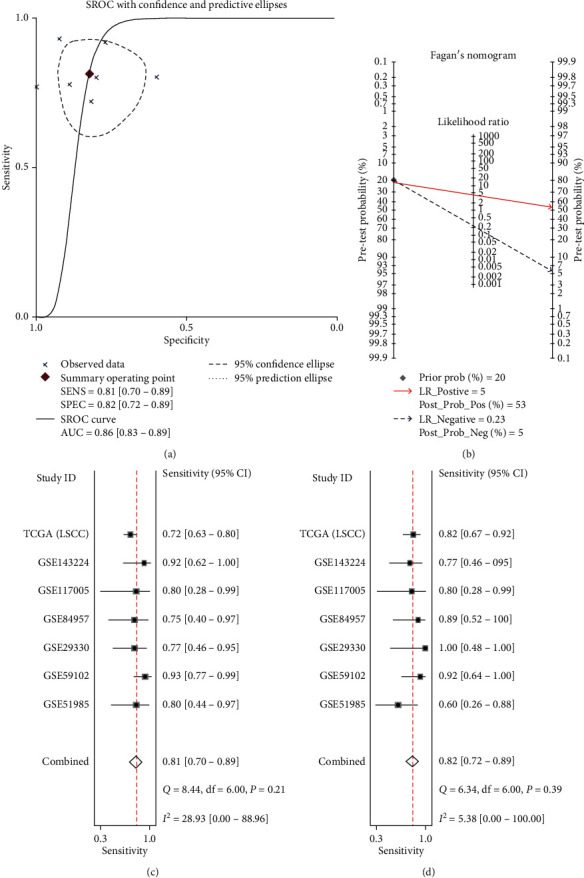
(a) The summary receiver operating characteristic curves analysis of IL24 mRNA for discriminating LSCC from noncancer tissues based on GEO and TCGA databases. (b) Prior probability and postprobability positive and negative of the included studies. The prior probability, postprobability positive, and postprobability negative reached 20%, 53%, and 5%, respectively (LR: likelihood ratio). (c, d) 0.81 (95% CI = 0.70−0.89) and 0.82 (95% CI = 0.72−0.89), respectively.

In this study, 21 microarrays were collected from the GEO database, and RNA-Seq data were extracted from TCGA database to analyze the IL24 mRNA expression between OSCC samples and noncancer samples. The details of microarrays and RNA-Seq data are shown in Table 2. Compared with noncancer tissues, all the data sets showed that IL24 was highly expressed in OSCC, and 18 of these data sets had statistical significance (*P* < 0.05). The abovementioned results were visualized by box diagrams and ROC curves (Figures 2(m), 3(a)–3(o), and 4(a)–4(f) in the supplementary materials).

To comprehensively analyze the IL24 mRNA expression in OSCC as a whole, all data sets were integrated and analyzed, and the results were combined into a forest plot, which showed that the IL24 mRNA was highly expressed in OSCC (SMD = 2.65, 95% CI = 1.66–3.63, *P* < 0.0001) (Figure 1(a)). The results showed heterogeneity, so the random effects model was used (*I*^2^ = 96.9%, *P* < 0.0001; Figure 1(b)). In addition, no significant publication bias was found (Begg's test, *P*=0.151; Egger's test, *P*=0.599, Figures 1(c) and 1(d)). Furthermore, the sROC curve was used to evaluate the ability of the IL24 mRNA to distinguish OSCC from noncancer samples, and the results suggested that the AUC was 0.94 (sensitivity = 0.92, specificity = 0.90), which demonstrates diagnostic ability (Figures 2(a), 2(c), and 2(d)). We also calculated the likelihood ratio to synthesize the reliability of the sensitivity and specificity. A positive likelihood ratio (+LR) of 9 indicated that the true positive rate of patients with OSCC diagnosed with a high expression of the IL24 mRNA was 9 times higher than the false positive rate. The negative likelihood ratio (−LR) was the ratio of the false negative rate to the true negative rate. A −LR of 0.09 indicated that the probability of a false negative judgment was 0.09 times that of a correct negative judgment (Figure 2(b)).

### 3.3. The Clinical Significance of the IL24 mRNA in LSCC by RNA-Seq

We collected 111 LSCC samples and the related clinical information from TCGA database. The IL24 mRNA was significantly higher expressed in LSCC tissues than in noncancer tissues (*P* < 0.0001, Figure 2(n) in the supplementary materials). We also calculated the clinical parameters of IL24 mRNA expression, such as age, gender, and pathological grade, in LSCC and noncancer tissues. However, no statistically significant difference was found. Similar to the nonsignificant influence of IL24 on the survival of OSCC, no remarkable correlation was noted between the IL24 expression and prognosis of LSCC patients (Figure 2(q) in the supplementary materials).

### 3.4. Analysis of IL24 mRNA Expression in LSCC by Microarrays and RNA-Seq Data

To analyze the IL24 mRNA expression in LSCC, we collected six microarrays from the GEO database and RNA-Seq data from TCGA database on LSCC (Table 2). The calculated results of each data set showed that the IL24 mRNA was highly expressed in LSCC, and six of these data sets were statistically significant (*P* < 0.05, Figures 2(a), 2(n), 4(k)–4(o) in the supplementary materials). Due to the existence of heterogeneity, the random effects model was used to combine each data set for the overall analysis, and the forest plot showed that the IL24 mRNA was highly expressed in LSCC (SMD = 1.77, 95% CI = 1.10–2.43, *P* < 0.0001, Figures 3(a) and 3(b)). No publication offset was found (Begg's test, *P*=0.133; Egger's test, *P*=0.221, Figures 4(c) and 4(d)). The sROC curve showed that the IL24 mRNA also had some ability to differentiate LSCC from noncancer tissue (AUC = 0.86, sensitivity = 0.81, specificity = 0.82, Figures 4(a), 4(c), and 4(d)). We also calculated the likelihood ratio, and the following results were obtained: +LR = 5 and −LR = 0.23 (Figure 4(b)).

### 3.5. Analysis of IL24 mRNA Expression in NPC by Microarrays

In this study, only four microarrays from the GEO database on the IL24 mRNA expression in NPC were collected (Table 2), and the relevant clinical parameter data were not collected. Therefore, only a simple analysis of NPC was conducted. All four microarrays showed that the IL24 mRNA was highly expressed in NPC, but only one was statistically significant (GSE53819, *P*=0.007, Figures 4(g)–4(j) in the supplementary materials).

**Figure 5:**
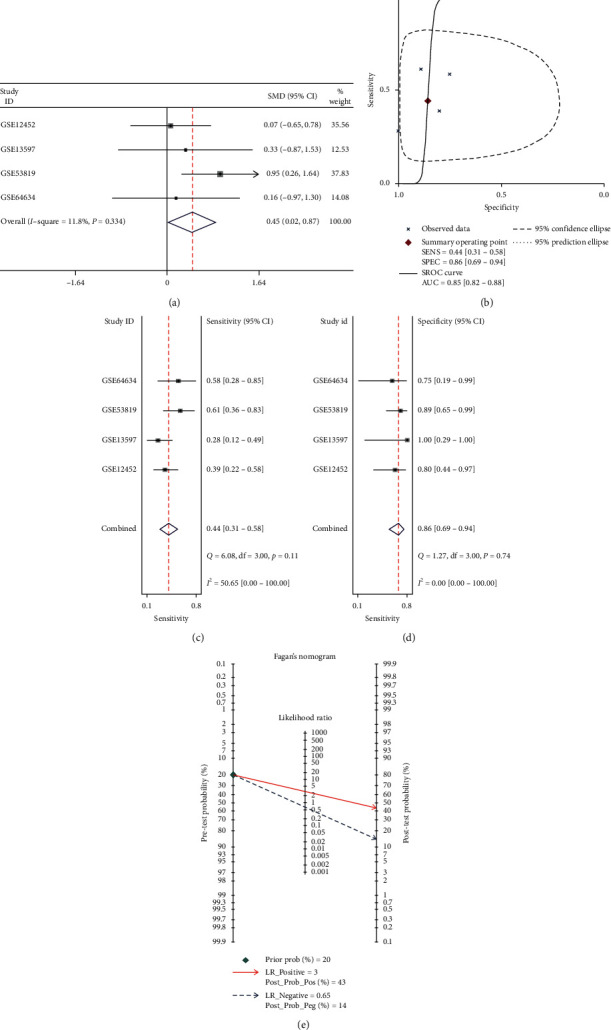
(a) The forest plot of 4 data sets evaluating the standard mean difference (SMD) of Interleukin 24 (IL24) mRNA expression between nasopharyngeal carcinoma (NPC) tissues and noncancer tissues based on Gene Expression Omnibus (GEO). (b) The summary receiver operating characteristic curves analysis of IL24 mRNA for discriminating NPC from noncancer tissues based on GEO database. (c, d) Sensitivity and specificity values of the included studies. The sensitivity and specificity values of the included studies were 0.44 (95% CI = 0.31−0.58) and 0.86 (95% CI = 0.69−0.94), respectively. (e) Prior probability and postprobability positive and negative of the included studies. The prior probability, postprobability positive, and postprobability negative reached 20%, 43%, and 14%, respectively (LR: likelihood ratio).

Using the fixed effects model (no heterogeneity, *I*^2^ = 11.8%, *P*=0.334), the forest plot showed that the IL24 mRNA was highly expressed in NPC (*P*=0.039, Figure 5(a)). The sROC curve showed the ability of the IL24 mRNA to distinguish NPC from noncancer (AUC = 0.85, sensitivity = 0.44, specificity = 0.86, Figures 5(b)–5(d)). The following results were also obtained: +LR = 3 and −LR = 0.65 (Figure 5(e)).

### 3.6. The Clinical Significance of the IL24 mRNA in HNSCC by RNA-Seq

In this study, a total of 502 HNSCC samples and the corresponding clinical data were collected from the TCGA database; these samples included 341 OSCC samples, 111 LSCC samples, and 40 tonsil cancer samples, among which the OSCC and LSCC subgroups were separately analyzed. The results showed that the IL24 mRNA was highly expressed in HNSCC compared to noncancer tissue (Figure 2(l) in the supplementary materials). Analyzing the clinical parameters, we found that IL24 mRNA expression was higher in patients with pathological grade 1–2 of HNSCC (Table 3). No relationship was observed between IL24 and the survival of HNSCC (Figure 2(o) in the supplementary materials).

### 3.7. Analysis of IL24 mRNA Expression in HNSCC by Microarrays and RNA-Seq Data

**Figure 6:**
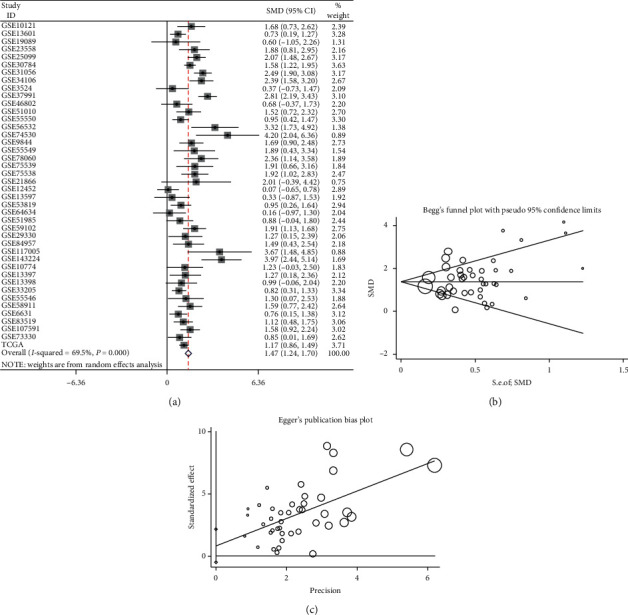
(a) The forest plot of 42 data sets. (b) Begg's funnel plot for the publication bias test. (c) Egger's funnel plot for the publication bias test.

**Figure 7:**
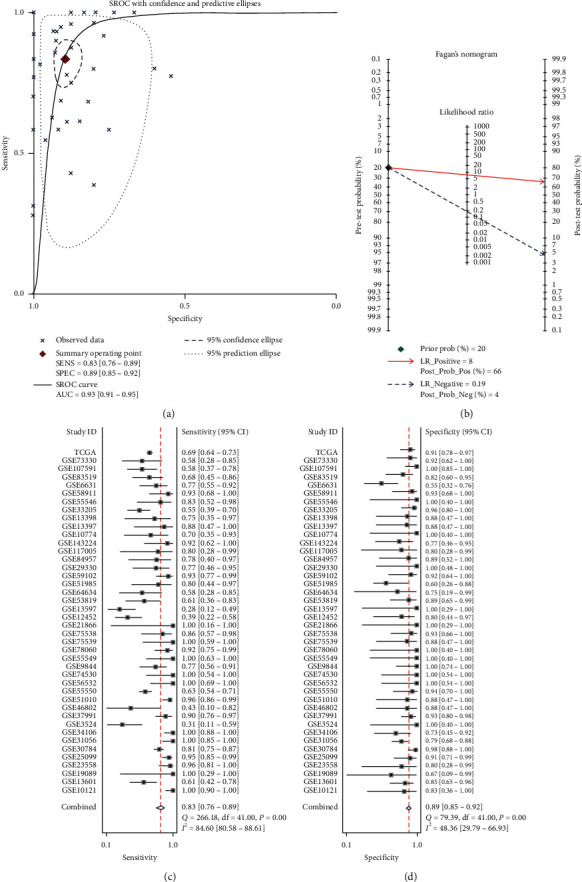
(a) The summary receiver operating characteristic curves analysis of IL24 mRNA for discriminating HNSCC from noncancer tissues based on GEO and TCGA databases. (b) Prior probability and postprobability positive and negative of the included studies. The prior probability, postprobability positive, and postprobability negative reached 20%, 66%, and 4%, respectively (LR: likelihood ratio). (c) and (d) Sensitivity and specificity values of the included studies. The sensitivity and specifcity were 0.83, 95% CI = 0.76–0.89 and 0.89, 95% CI = 0.85–0.92, respectively.

From the GEO database, we screened and collected a total of 41 microarrays for this study, including 21 microarrays of OSCC, 6 microarrays of LSCC, 4 microarrays of NPC, and 10 microarrays of HNSCC with an unclassified subgroup. The RNA-Seq data of HNSCC (including tonsil, oral cavity, hypopharynx, and larynx samples) were obtained from the TCGA database (Table 2). The analysis showed that the IL24 mRNA was highly expressed in all data sets, among which 31 data sets had statistically significant results (Figures 2(a)–2(l), 3(a)–3(o), and 4(a)–4(o) in the supplementary materials). To comprehensively analyze IL24 mRNA expression in HNSCC, we combined all the collected data and visualized the calculated results with a forest plot. Because of the heterogeneity (*I*^2^ = 69.5%, *P* < 0.0001), the random effects model was used. The results showed that the IL24 mRNA was highly expressed in HNSCC compared with noncancer tissues (SMD = 1.47, 95% CI = 1.24–1.70, Figure 6(a)), and there was no publication bias (Begg's test, *P*=0.176; Egger's test, *P*=0.201; Figures 6(b) and 6(c)). In addition, we constructed an sROC curve, whose AUC demonstrated the ability of IL24 mRNA expression to distinguish HNSCC from noncancer tissue (AUC = 0.93, 95% CI = 0.91−0.95, Figure 7(a)). The sensitivity and specificity were 0.83, 95% CI = 0.76−0.89 and 0.89, 95% CI = 0.85−0.92, respectively (Figures 7(c) and 7(d)). The following results were also obtained: +LR = 8 and −LR = 0.19 (Figure 7(b)).

## 4. Discussion

IL24 mRNA is known to have an apoptotic effect on cancer cells but not on noncancer cells [22, 23]. IL24 mRNA could induce autophagy as a protective mechanism, while 3-methyladenine (3-MA) inhibition of autophagy significantly enhanced the anticancer effect of IL24 in OSCC [18]. There also was a study that showed that the transfection of miR-205 into human oral carcinoma (KB) cells strongly induced the IL24 mRNA, which targeted the IL24 promoter directly to induce gene expression and had significant therapeutic potential to turn on silenced tumor suppressor genes [24]. Adenovirus-mediated IL24 mRNA therapy could enhance the in vivo and in vitro antitumor effects of radiotherapy for NPC [20, 21]. A study of *Bifidobacterium* as a carrier of the IL24 mRNA to treat HNSCC in mice showed that the recombinant strain *Bifidobacterium breve*-IL24 had a stronger ability to inhibit tumor growth and induce tumor cell apoptosis [19]. Although there is no suitable way to conduct clinical trials of the IL24 mRNA as a targeted therapy for HNSCC and its subgroups, the current studies suggest that the IL24 mRNA could be a potential treatment. The body will be protected by regulating the expression of the IL24 mRNA as an immune factor during cancer. Many studies have found that IL24 can be expressed through a variety of immune cells—especially *T*-helper cells (Th)2 cells and tissue cells, including keratinocytes, epithelial cells, and endothelial cells, and plays an important role in host defense, tissue protection, and autoimmune response regulation [25, 26]. It has also been studied in many cancers, such as prostate cancer, kidney cancer, osteosarcoma, melanoma, and colon cancer [13, 14, 16]. Induction of endoplasmic reticulum (ER) stress is one of the main mechanisms by which IL24 mRNA induces cell death. Compared with normal cells, cancer cells have higher levels of ER stress, which makes them more susceptible to ER stress-mediated cell death triggered by IL24 mRNA. Another possible explanation is that IL24 mRNA mediates different cell killing effects between normal and cancer cells depending on reactive oxygen species (ROS), and a number of studies have shown that cancer cells have higher levels of basic ROS than normal cells. Therefore, compared with normal cells, drugs such as IL24mRNA that enhance ROS production can overcome the natural antioxidant effect in cancer cells more effectively, leading to cell death [9, 27]. However, neither the correlation between IL24 mRNA expression and HNSCC has been analyzed in the literature nor has there been a detailed study of IL24 mRNA expression in HNSCC and its subgroups or a cross-sectional comparison between them. Therefore, this study mainly analyzed IL24 mRNA expression and the corresponding clinical significance in HNSCC and its subgroups at the mRNA level.

HNSCC is a squamous cell tumor composed of various subgroups, among which the most common subgroups are OSCC, NPC, and LSCC [28, 29]. Therefore, we mainly analyzed these three common subgroups and HNSCC as a whole group in this study. To more accurately illustrate the clinical significance of the IL24 mRNA in HNSCC and its subgroups, substantial IL24 mRNA expression data were selected by searching several databases. We ultimately collected a total of 41 microarrays and RNA-seq on HNSCC from the GEO and TCGA databases. Among them, 21 were for OSCC; 4 were for NPC; and 6 were for LSCC; the others were for an unclassified subgroup of HNSCC. A total of 1,564 HNSCC tissue samples (including 1060 cases of OSCC, 86 cases of NPC, and 181 cases of LSCC) and 603 noncancer tissue samples, as well as the corresponding clinical information regarding 502 HNSCC patients (including 341 cases of OSCC and 111 cases of LSCC), were included. The inclusion of many samples made the results of this analysis more reliable and more detailed. We utilized the microarrays and RNA-seq data to analyze the difference in IL24 mRNA expression in each subgroup and HNSCC compared with noncancer tissue. Then, we investigated the relationship between IL24 mRNA expression and clinical parameters. The results showed that the IL24 mRNA was highly expressed in HNSCC and its subgroups and had the ability to distinguish between cancer and noncancer tissues. In HNSCC and its subgroups, the results showed that these patients who highly expressed the IL24 mRNA seemed to have a better prognosis.

In this study, the IL24 mRNA had significantly higher expression in OSCC, as well as in LSCC, compared to noncancer tissues, and both comparisons had significant statistical differences (*P* < 0.0001). The sROC curve showed the AUC of OSCC was 0.94, while the AUC of LSCC was 0.86, which both suggested the IL24 mRNA had the ability to distinguish between tumor tissues and noncancer tissues. In addition, we also studied the relevant clinical parameters of IL24 mRNA expression in OSCC and LSCC, such as age, gender, and pathological stage, but did not find statistically significant indicators. Then, we conducted a subgroup analysis of NPC. Compared with other types of HSNCC, the susceptibility factors leading to NPC somewhat differed. A common factor is Epstein–Barr virus (EBV) infection, and a rare factor is HPV infection; although some studies have reported cases, there is no significant correlation between HPV infection and NPC [30–32]. We found that IL24 was highly expressed in NPC (*P*=0.039). These findings suggest that NPC, like other HNSCC tissues, may be related to high expression of the IL24 mRNA, which is worth further study of the molecular mechanism in the future.

We combined all the collected data to analyze IL24 mRNA expression in HNSCC as a whole. IL24 was highly expressed in HNSCC and had the ability to distinguish HNSCC from noncancer tissue. Analyzing the clinical parameters, we found that IL24 mRNA expression was higher in patients with pathological grade 1–2 of HNSCC. IL24 was reported to be a potential prognostic biomarker and an indicator of malignancy in HNSCC in 2014 [33]. In vitro tests showed that high expression of mda-7/IL24 can upregulate the expression of the epithelial terminal differentiation markers cytokeratin (KRT) 1, KRT4, KRT13, and phosphorylated endoplasmic reticulum stress protein (p)-EIF2a. However, it also may be part of the underlying mechanism involved in mda-7-mediated HNSCC differentiation and apoptosis via downregulation of the epithelial proliferative markers KRT5, KRT14, integrin *β*4, and antiapoptosis protein Bcl-2. Although this study analyzed the potential pathway mechanism of high expression of the IL24 mRNA in HNSCC as a whole part, no other study has conducted the same analysis for the IL24 mRNA in a subgroup of HNSCC. Therefore, we mainly analyzed the expression of the IL24 mRNA in OSCC, LSCC, and NPC, which was the same as that in HNSCC, and they all demonstrated high IL24 mRNA expression. This finding also suggested that the expression mechanism of the IL24 mRNA in each subgroup may be the same as that in HNSCC, which is worth a detailed study in the future to provide more accurate targeted therapy candidates for each subgroup of HNSCC. In addition, we analyzed the Kaplan–Meier curves in HNSCC and each subgroup, all of which suggested that IL24 mRNA expression had no significant correlations with the status of prognosis, which needs to be verified with more cohorts. IL24 can protect the body via the induction of tumor cell apoptosis and inhibition of angiogenesis [10, 11] in cancers such as colorectal adenocarcinoma and hepatic carcinoma [34, 35]. IL24 has also been found in many diseases involving inflammation, such as cardiovascular disease, rheumatoid arthritis, *tuberculosis*, and viral infections, and may play a role in psoriasis [15, 36]. In addition, many studies have shown that IL24 mRNA can be used as a potential tumor treatment [37, 38]. All these characteristics suggest that the IL24 mRNA may provide a potential idea for the treatment of HNSCC and its subgroups, which is significant for further research with a larger sample size.

However, this study also had some limitations. There was a large difference in the number of samples between cancer and noncancer. Although appropriate statistical methods were used for analysis, the error of calculation results cannot be completely avoided. We did not further study the molecular mechanism and pathway of IL24 mRNA expression in each subgroup, which is worth further study in the future. The expression level of the IL24 mRNA in the bodily fluid of patients with HNSCC represents its potential for early diagnosis in clinical settings, which also requires further testing.

In conclusion, the IL24 mRNA was highly expressed in HNSCC, as well as in its subgroups—OSCC, LSCC, and NPC. According to the results of sROC, IL24 mRNA also had the ability to distinguish between cancer and noncancer tissues in HNSCC and its subtypes, respectively. The IL24 mRNA level may be used as a potential marker for HNSCC screening and was worthy of further verification in the future. We hope that analysis of the IL24 mRNA expression pathway and molecular mechanism will be conducted and explored the relationship of the IL24 mRNA with the development of HNSCC and disease progression with a larger sample size. In addition, we hope to provide a new idea for the treatment of the IL24 mRNA in HNSCC and its subgroups.

## Figures and Tables

**Table 1:** The relationships between IL24 mRNA levels and clinicopathologic features of the 341 cases of OSCC patients detected by RNA-sequencing.

Parameters	*n*	Mean	±SD	*t*	*P* value
Tissues					
OSCC	341	4.055	±0.2013	10.973	<0.0001
Non-OSCC	44	3.808	±0.1306		

Gender					
Male	238	4.047	±0.2134	−1.172	0.242
Female	103	4.073	±0.1694		

Age					
≥60	190	4.052	±0.2054	−0.268	0.789
＜60	150	4.058	±0.1971		

Grade					
G1-G2	260	4.068	±0.2006	−1.767	0.078
G3-G4	72	4.021	±0.1946		

Pathological stage					
I-II	92	4.065	±0.1955	0.598	0.55
III-IV	239	4.051	±0.2048		

Pathological T stage					
T1-T2	130	4.047	±0.1889	−0.544	0.587
T3-T4	208	4.059	±0.2095		

Pathological N stage					
N0	173	4.056	±0.1883	0.358	0.721
N1	154	4.048	±0.2175		

Pathological M stage					
M0	320	4.053	±0.2019	0.881	0.379
M1	2	3.927	±0.3059		

Note: OSCC: oral squamous cell carcinoma; G: grade; T: tumor; N: lymph node; M: metastasis.

**Table 2:** The basic information of the 39 microarrays and RNA-Seq datasets of IL24 mRNA expression profiling.

ID	Subgroup	Author	Country	HNSCC tissue			Non-HNSCC tissue			*P* value
				*N*	Mean	±SD	*N*	Mean	±SD	
GSE10121	OSCC	Sticht C	Germany	34	3.027	±1.612	6	0.404	±1.194	0.001
GSE13601	OSCC	Singh B	USA	31	5.611	±1.165	26	4.859	±0.833	0.007
GSE19089	OSCC	Smith DI	USA	3	6.181	±0.223	3	5.931	±0.541	0.501
GSE23558	OSCC	Ambatipudi S	India	27	4.374	±2.128	5	0.437	±1.865	0.001
GSE25099	OSCC	Peng C	Taiwan	57	6.368	±1.275	22	4.073	±0.395	<0.0001
GSE30784	OSCC	Chen C	USA	167	6.117	±2.382	45	2.739	±0.517	<0.0001
GSE31056	OSCC	Reis PP	USA	23	6.178	±2.715	73	2.395	±0.875	<0.0001
GSE34106	OSCC	Rentoft M	UK	28	9.388	±0.541	15	7.467	±1.152	<0.0001
GSE3524	OSCC	Toruner GA	Sweden	16	−0.431	±1.845	4	−1.109	±1.799	0.517
GSE37991	OSCC	Lee CH	USA	40	11.701	±0.983	40	7.1599	±2.065	<0.0001
GSE46802	OSCC	Towle R	Canada	7	1.261	±1.723	8	0.446	±0.368	0.213
GSE51010	OSCC	Saeed AA	UK	48	6.451	±0.873	8	5.203	±0.271	<0.0001
GSE55550	OSCC	Tomar S	USA	139	0.444	±2.573	16	−1.909	±1.471	<0.0001
GSE56532	OSCC	Pavuluri S	Australia	10	8.873	±1.033	6	6.084	±0.229	<0.0001
GSE74530	OSCC	Oghumu S	USA	6	9.416	±1.842	6	3.892	±0.259	0.001
GSE9844	OSCC	Zhou X	USA	26	7.201	±1.977	12	4.397	±0.362	<0.0001
GSE55549	OSCC	Tomar S	USA	8	2.203	±2.537	4	−1.828	±0.477	0.012
GSE78060	OSCC	Enokida T	Japan	26	10.225	±1.849	4	5.969	±1.337	<0.0001
GSE75539	OSCC	Krishnan NM	India	7	7.463	±0.859	8	6.295	±0.245	0.003
GSE75538	OSCC	Krishnan NM	India	14	10.257	±1.482	14	7.646	±1.219	<0.0001
GSE21866	OSCC	Ceder R	Sweden	2	4.544	±0.479	3	1.032	±2.108	0.114
GSE12452	NPC	Ahlquist P	USA	31	5.739	±0.338	10	5.719	±0.142	0.854
GSE13597	NPC	Yap L	UK	25	2.569	±1.963	3	1.92	±1.619	0.591
GSE53819	NPC	Bao YN	China	18	6.672	±2.014	18	5.214	±0.808	0.007
GSE64634	NPC	Xiong W	China	12	5.511	±1.139	4	5.339	±0.648	0.783
GSE51985	LSCC	Fang J	China	10	5.382	±1.781	10	3.886	±1.619	0.065
GSE59102	LSCC	Bueno RB	Brazil	29	7.166	±2.084	13	3.503	±1.466	<0.0001
GSE29330	LSCC	Demokan S	USA	13	6.392	±2.366	5	3.737	±0.777	0.028
GSE84957	LSCC	Feng L	China	9	5.276	±1.888	9	3.156	±0.707	0.011
GSE117005	LSCC	Liu M	China	5	9.836	±0.285	5	8.775	±0.294	0.032
GSE143224	LSCC	Nicolau-Neto PP	Brazil	14	6.569	±0.239	11	5.877	±0.048	0.019
GSE10774	Unclassified	Yang X	USA	10	0.497	±0.302	4	0.153	±0.195	0.059
GSE13397	Unclassified	Qi Y	USA	8	7.156	±1.368	8	5.709	±0.850	0.024
GSE13398	Unclassified	Qi Y	USA	8	1.426	±1.127	8	0.531	±0.599	0.067
GSE33205	Unclassified	Ochs MF	USA	44	5.997	±1.094	25	5.255	±0.398	<0.0001
GSE55546	Unclassified	Tomar S	USA	12	1.004	±1.998	4	−1.309	±0.474	0.041
GSE58911	Unclassified	Lobert S	USA	15	7.451	±1.547	15	5.693	±0.195	0.001
GSE6631	Unclassified	Kuriakose MA	USA	22	4.422	±0.449	22	4.152	±0.216	0.015
GSE83519	Unclassified	Martens-de Kemp SR	Netherlands	22	0.947	±0.437	22	0.449	±0.454	0.001
GSE107591	Unclassified	Blandino G	Italy	24	6.034	±1.726	23	4.049	±0.339	<0.0001
GSE73330	Unclassified	Chen P	USA	12	4.579	±0.464	12	4.281	±0.172	0.055
TCGA	HNSCC	—	USA	502	4.035	±0.197	44	3.808	±0.131	<0.0001
	OSCC	—	USA	341	4.055	±0.011	44	3.808	±0.131	<0.0001
	LSCC	—	USA	111	4.003	±0.191	44	3.808	±0.131	<0.0001

Note: head and neck squamous cell carcinoma (HNSCC); standard deviation (SD); oral squamous cell carcinoma (OSCC); nasopharyngeal squamous cell carcinoma (NPC); laryngeal squamous cell carcinoma (LSCC).

**Table 3:** The relationships between IL24 mRNA levels and clinicopathologic features of the 502 cases of HNSCC patients detected by RNA-sequencing.

Parameters	*n*	Mean	±SD	*t*	*P* value
Tissue					
HNSCC	502	4.035	±0.1973	10.504	<0.0001
Non-HNSCC	44	3.808	±0.1306		

Gender					
Male	368	4.026	±0.2058	−1.853	0.065
Female	134	4.059	±0.1704		

Age					
≥60	256	4.031	±0.2038	−0.347	0.729
<60	245	4.037	±0.2039		

Grade					
G1-G2	362	4.053	±0.1961	2.771	0.006
G3-G4	121	3.996	±0.1891		

Pathological stage					
I-II	114	4.051	±0.2025	1.047	0.296
III-IV	374	4.028	±0.1964		

Pathological T stage					
T1-T2	177	4.027	±0.1909	−0.561	0.575
T3-T4	310	4.037	±0.2021		

Pathological N stage					
N0	239	4.037	±0.1894	0.643	0.521
N1	241	4.025	±0.2061		

Pathological M stage					
M0	472	4.032	±0.1975	−0.112	0.911
M1	5	4.042	±0.2353		

Note: HNSCC: head and neck squamous cell carcinoma; G: grade; T: tumor; N: lymph node; M: metastasis.

## Data Availability

Data used for analysis were extracted from the publicly available databases—the Gene Expression Omnibus (GEO) and the Cancer Genome Atlas (TCGA) databases. The information of microarrays and RNA-Seqs data used to support the findings of this study are included within article. Please see Table 2 for details.

## Supplementary Materials

Supplementary Materials Figure 1: flow chart of the study design. Supplementary materials: Figure 2: (a) the expression data of IL24 mRNA and the corresponding ROC curves in LSCC tissues from GEO database. (b–k) The expression data of IL24 mRNA and the corresponding ROC curves in unclassified subgroups HNSCC tissues in 10 microarrays from GEO database. (l–n) The expression data of IL24 mRNA and the corresponding ROC curves in HNSCC and its subgroups (OSCC and LSCC) tissues in RNA‐Seq by TCGA database. (o) The Kaplan–Meier survival curve of IL24 mRNA in the HNSCC by RNA‐Seq. (p) The Kaplan–Meier survival curve of IL24 mRNA in the OSCC by RNA-Seq. (q) The Kaplan–Meier survival curve of IL24 mRNA in the LSCC by RNA-Seq.Note: receiver operating characteristic (ROC); head and neck squamous cell carcinoma (HNSCC); oral squamous cell carcinoma (OSCC); laryngeal squamous cell carcinoma (LSCC). Figure 3: (a–o) the expression data of IL24 mRNA and the corresponding ROC curves in OSCC tissues in 15 microarrays from the GEO database, Figure 4: (a–f) the expression data of IL24 mRNA and the corresponding ROC curves in OSCC tissues in 6 microarrays from GEO database. (g–j) The expression data of IL24 mRNA and the corresponding ROC curves in NPC tissues in 4 microarrays from GEO database. (k–o) The expression data of IL24 mRNA and the corresponding ROC curves in LSCC tissues in 5 microarrays from GEO database.
