# Supplementary material for: Clinical Significance of the Interleukin 24 mRNA Level in Head and Neck Squamous Cell Carcinoma and Its Subgroups: An In Silico Investigation
Source: J Oncol. 2020 Sep 18;2020:7042025. doi: 10.1155/2020/7042025 (PMC7519990; doi:10.1155/2020/7042025)
Supplement: Supplementary Materials — Figure 1: flow chart of the study design. Supplementary materials: Figure 2: (a) the expression data of IL24 mRNA and the corresponding ROC curves in LSCC tissues from GEO database. (b–k) The expression data of IL24 mRNA and the corresponding ROC curves in unclassified subgroups HNSCC tissues in 10 microarrays from GEO database. (l–n) The expression data of IL24 mRNA and the corresponding ROC curves in HNSCC and its subgroups (OSCC and LSCC) tissues in RNA‐Seq by TCGA database. (o) The Kaplan–Meier survival curve of IL24 mRNA in the HNSCC by RNA‐Seq. (p) The Kaplan–Meier survival curve of IL24 mRNA in the OSCC by RNA-Seq. (q) The Kaplan–Meier survival curve of IL24 mRNA in the LSCC by RNA-Seq.Note: receiver operating characteristic (ROC); head and neck squamous cell carcinoma (HNSCC); oral squamous cell carcinoma (OSCC); laryngeal squamous cell carcinoma (LSCC). Figure 3: (a–o) the expression data of IL24 mRNA and the corresponding ROC curves in OSCC tissues in 15 microarrays from the GEO database, Figure 4: (a–f) the expression data of IL24 mRNA and the corresponding ROC curves in OSCC tissues in 6 microarrays from GEO database. (g–j) The expression data of IL24 mRNA and the corresponding ROC curves in NPC tissues in 4 microarrays from GEO database. (k–o) The expression data of IL24 mRNA and the corresponding ROC curves in LSCC tissues in 5 microarrays from GEO database. [file 7042025.f1.pdf]

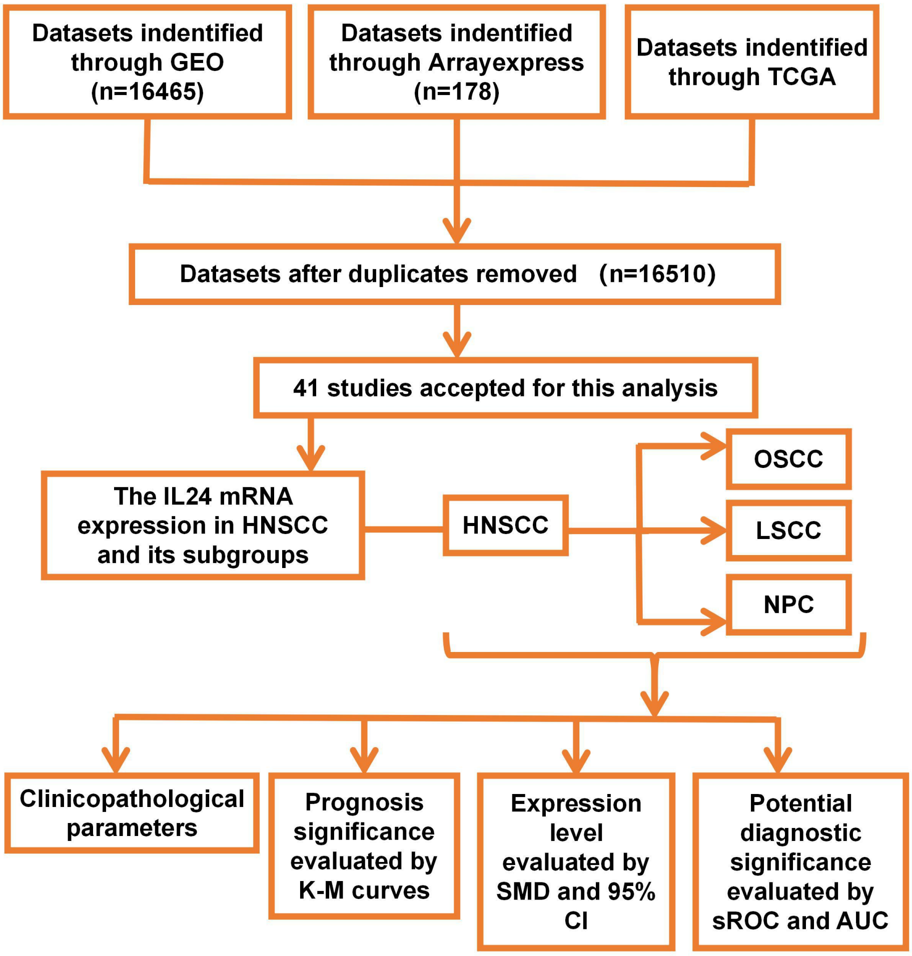


Supplementary materials: Figure 1 Flow chart of the study design.

Note：head and neck squamous cell carcinoma (HNSCC); nasopharyngeal carcinoma (NPC); laryngeal squamous cell carcinoma (LSCC); oral squamous cell carcinoma (OSCC); Kaplan-Meier (K-M); standard mean difference (SMD); confidence interval (CI); summary receiver operating characteristic (sROC); area under the curve (AUC).

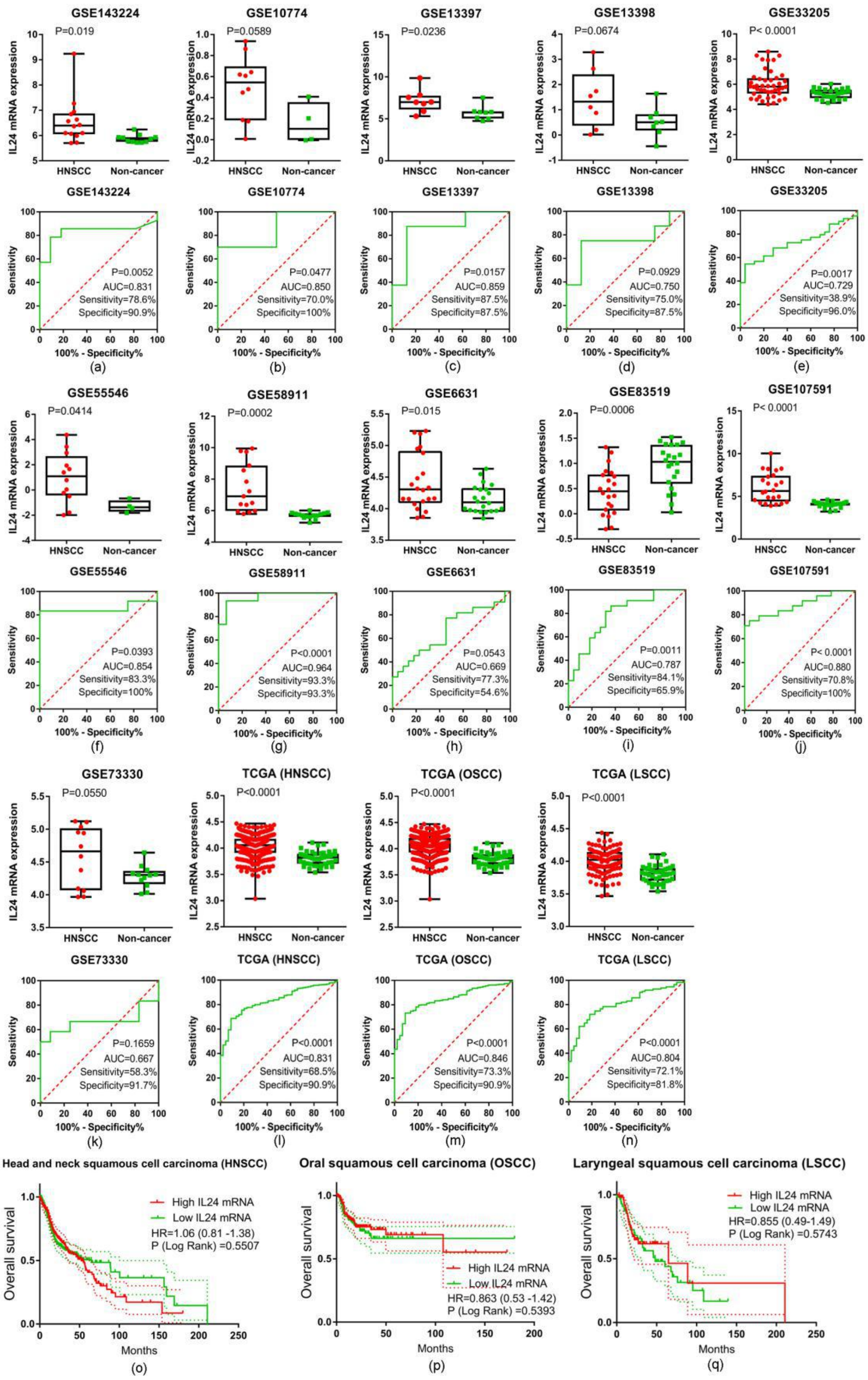


Supplementary materials: Figure 2.

(a): The expression data of IL24 mRNA and corresponding the ROC curves in LSCC tissues from GEO database;

(b-k): The expression data of IL24 mRNA and corresponding the ROC curves in unclassified subgroups HNSCC tissues in 10 microarrays from GEO database.

(l-n)： The expression data of IL24 mRNA and corresponding the ROC curves in HNSCC and its subgroups (OSCC and LSCC) tissues in RNA-Seq by TCGA database.

(o): The Kaplan-Meier survival curve of IL24 mRNA in the HNSCC by RNA-Seq

(p): The Kaplan-Meier survival curve of IL24 mRNA in the OSCC by RNA-Seq

(q): The Kaplan-Meier survival curve of IL24 mRNA in the LSCC by RNA-Seq

Note： receiver operating characteristic (ROC); head and neck squamous cell carcinoma (HNSCC); oral squamous cell carcinoma (OSCC); laryngeal squamous cell carcinoma (LSCC);

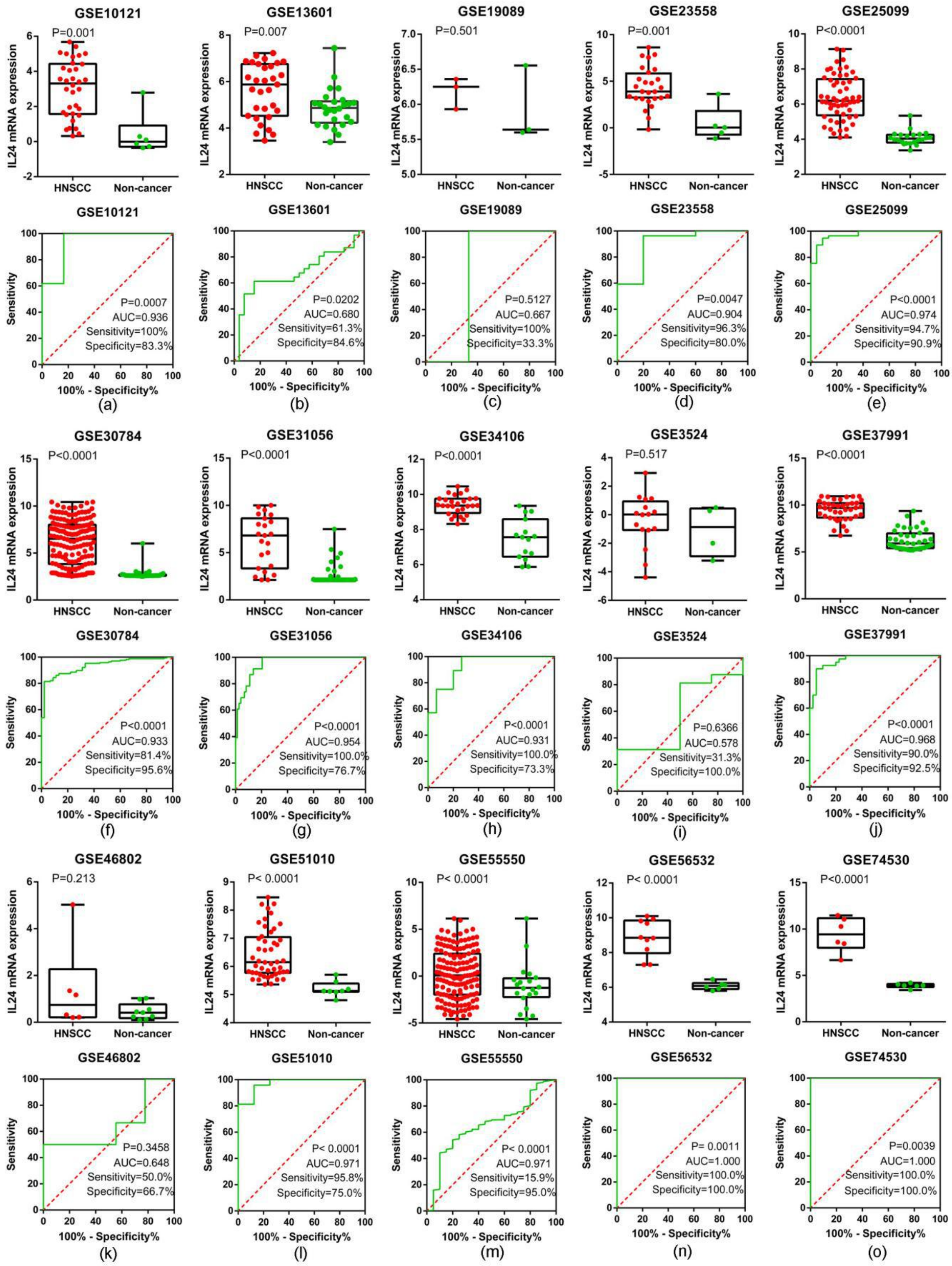


Supplementary materials: Figure 3:

(a-o): The expression data of IL24 mRNA and corresponding the ROC curves in OSCC tissues in 15 microarrays from GEO database;

Note：receiver operating characteristic (ROC), oral squamous cell carcinoma (OSCC)

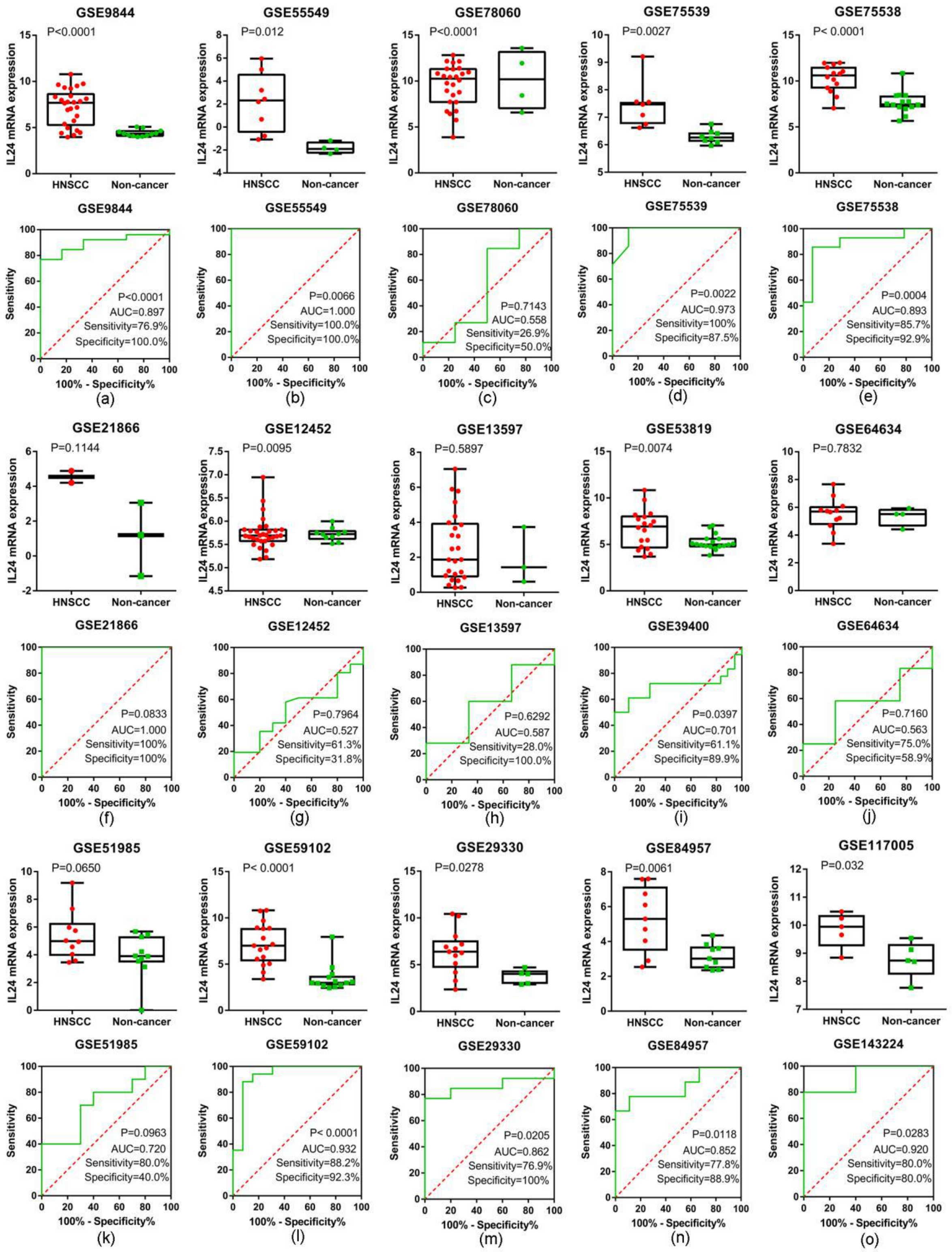


Supplementary materials: Figure 4:

(a-f): The expression data of IL24 mRNA and corresponding the ROC curves in OSCC tissues in 6 microarrays from GEO database;

(g-j): The expression data of IL24 mRNA and corresponding the ROC curves in NPC tissues in 4 microarrays from GEO database;

(k-o): The expression data of IL24 mRNA and corresponding the ROC curves in LSCC tissues in 5 microarrays from GEO database;

Note： receiver operating characteristic (ROC); oral squamous cell carcinoma (OSCC); nasopharyngeal carcinoma (NPC); laryngeal squamous cell carcinoma (LSCC);
